# Rethinking global mental health and its priorities

**DOI:** 10.1017/S204579601900060X

**Published:** 2019-10-11

**Authors:** B. Saraceno

**Affiliations:** Lisbon Institute of Global Mental Health, Lisbon, Portugal

**Keywords:** Global mental health, low-income countries, setting priorities

## Abstract

Global mental health (GMH) seems to enjoy increasing visibility in the global health and development discourse. However, this visibility implies also the urgency of addressing few questions about new priority setting in the domains of policy, care delivery, service organisation and research. Even before trying to answer these questions, rethinking more deeply the notion and implications of GMH seems to be a useful collective exercise. Some unanswered questions should be at the core of this exercise: Is GMH really global or rather Western? Is GMH concerned enough with local context? Is GMH too unbalanced towards a biomedical model? What are the consequences of the predominant emphasis given by GMH on common mental disorders and primary care level on people with severe mental disabilities? GMH is not global but rather it is hegemonised by western institutions. It would be useful to have an independent and very inclusive think tank which should promote a global debate on these issues and offer an unbiased support to WHO.

## Introduction

Global mental health (GMH) seems to enjoy increasing visibility in the global health and development discourse. We see an amazing and growing interest of academia which multiplies summer courses, masters, diplomas and of course, grant applications. The simple presence of the words ‘mental health’ in few lines of the large UN Sustainable Development Goals document has created an immense excitement. WHO has pursued in a coherent way the cycle initiated in the year 2001 with the World Health Report (WHO, 2001) devoted to mental health with several and useful documents and guidelines culminating in 2013 with the important Mental Health Action Plan 2013–2020 endorsed by all member states (WHO, 2013).

With the goal of putting mental health at the centre of global health and development priorities, the World Bank Group and WHO co-hosted the meeting ‘Out of the Shadows: Making Mental Health a Global Priority’ in Washington, DC (World Bank Group/International Monetary Fund spring meetings 13 and 14 April 2016, Washington, DC). This event aimed to increasing awareness about mental health as a development challenge and the associated economic and social costs of inaction. Following the first Global Summit held in Los Angeles, in May 2017, a second Global Summit on Mental Health Culture Change, was hosted in London by the UK government (Global Ministerial Mental Health Summit, 11–12 October 2018, London). The London Summit gathered together outstanding scholars, some government officials and stakeholders, to focus on removing cultural biases that affect how people think and talk about mental health. The London Summit was also the occasion for the launching of the Lancet Commission on Global Mental Health (Patel *et al.*, 2018). Indeed, it seems that GMH has entered a golden time. However, this visibility implies also the urgency of addressing a few questions about new priority setting in the domains of policy, care delivery, services organisation and research.

## Challenging questions

Nevertheless, even before trying to answer these questions, rethinking more deeply the notion and the implications of GMH seems to be a useful collective exercise.

Indeed, some challenging questions must be at the core of this exercise which ideally should be run as a candid and unbiased public debate.

- Is GMH really global or rather Western?
- Is GMH concerned enough with local context?
- Is GMH too unbalanced towards a biomedical model?
- What are the consequences of the predominant emphasis given by GMH on common mental disorders and primary care level on people with severe mental disabilities and on the human rights of people in psychiatric institutions?

Answering these questions would be not easy at all. A large public debate is needed, and it should involve different stakeholders, including professionals from the field, experts who are not part of the academic community and do not operate just in the high-income countries but come from the Global South.

There is no doubt that today the leadership of the global mental discourse is seating in few western academic centres (with the very positive exception of South Africa). The language used is exclusively English. Few outstanding academic centres propose themselves as the hub for all GMH activities: from thinking to distributing money. A recent paper (Vigo *et al*., 2019) proposes a multi-sectoral and multi-organisational model of partnership to raise and allocate funds and to support countries to use funds effectively. Even if the authors feel the need to be reassuring: ‘…a decision-making process that is based on genuine participation, avoid the emergence of dominion partners…’ some concerns about an unbalanced power are legitimate.

In addition, the establishment of a countdown group responsible for monitoring GMH indicators is announced by Lancet (Saxena *et al*., 2019). The countdown will involve Harvard, Lancet, the Movement for Global Mental Health, the organisation United for Global Mental Health and, hopefully, the WHO.

Looking carefully at all these initiatives and organisations it appears quite evident that the same centres are everywhere, becoming influencers and positioning themselves as a dominant group. Therefore, it is vital that WHO would not become a passive or just a minority partner; WHO must keep its convening power, its moral authority, independency and overall its ability to speak to the world staying above the emergence of any cultural and technical hegemony: this is the ultimate philosophy of multilateralism.

It is for these reasons that the answer to the first question is probably ‘No’, namely, Global Mental Health, is not global but rather it is hegemonised by western institutions.

The overwhelming influence of academic centres based in western high-income countries is reflected by the scarce attention given by the GMH discourse to local contexts. According to Bracken *et al*. (2016), ‘Currently one quarter of the global population lives in near destitution and 3.5 million children die of starvation annually. What is “mental health” in this broken social world?’ (Summerfield, 2012). An approach to mental health that does not see contextual issues as primary and that ‘promotes a reductionist understanding of mental illness has the potential to do a great deal of harm’ (Summerfield, 2012). Nevertheless, interventions tend to be limited to psychotropic medication, cognitive behaviour therapy and interpersonal therapy or variations of these (Patel *et al*., 2007). However, successful programmes for common mental disorder may need to deal with the social and economic issues that people experience while at the same time addressing the mental health issues – as for example Basic Needs (http://www.basicneeds.org/) does with people with severe mental disorder. In too many cases symptom alleviation is a laudable outcome, but in other instances this may merely help to cover up an underlying dysfunctional (personal or social) situation (Freeman, 2016).

Context and the impact of social determinants seem to be factors considered by theoretical modelling, (Patel *et al.*, 2018) but too often they remain absent from the practical strategies and interventions promoted in low- and middle-income countries. GMH is essentially promoting a psycho-biomedical model where social factors are not part of the interventions. We all have enthusiastically endorsed the notion of closing or reducing the gap between untreated and treated and therefore, the urgent need of scaling-up mental health care. However, we have to build the ‘content’ of the scaling-up: what actually do we want to scale-up?

If we do not put a clear content to this process, we will certainly get a sort of easy and general consensus about scaling-up and reducing the gap, but this type of consensus may be too generic.

Let's be clear: in scaling-up mental health care are we scaling-up outmoded psychiatric hospitals (as many psychiatrists would like)? Compulsive hospitalisation with different rules and rights depending on the gender of the patient (as some countries do)? Unmodified electroshocks ‘exceptionally allowed’ (as some professionals seem to recommend); finally, are we scaling-up also the unacceptable influence of Big Pharma on psychiatric prescribing practices? Of course, the answer should be: No, we do not want to scale-up all this, but we must say this more clearly and make our efforts to scaling-up care more comprehensive and inclusive of innovative policies and human rights driven mental health service organisations. For sure, we need a more trans-disciplinary approach and opening dialogues and collaborations with service users, we need to look at legislations and their coherence with international covenants on human rights, we need to look at the social and economic rights of people with mental disorders and disability as recommended by the UN, the Inter American and the European charters and conventions.

## Broadening the notion of scaling-up

In other words, we cannot simply try to ‘export’ treatment packages even if they are of good quality and evidence based: this is no longer enough.

We should be concerned about the increasing trend of GMH discourse to reduce the notion of scaling-up just to treatments as it would be possible to provide treatments in a vacuum instead than in a policy framework. In other words, the term ‘global’ should be seen not just as a geographical extension of countries which are passive recipients of technical cooperation in the narrow area of psychiatric treatments but rather, as the promotion of comprehensive mental health policies and care provision.

In conclusion, the Executive Board of the Consortium of Universities for Global Health (Koplan *et al*., 2009) defines Global Health as an area for study, research and practice that places a priority on improving health and achieving equity in health for all people worldwide and emphasises transnational health issues, social determinants and solutions; Global Health involves many disciplines within and beyond the health sciences and should promote interdisciplinary collaboration. In other words, Global Health goes much beyond medical paradigm and introduces the key notion and the goal of improving health and achieving equity in health using a comprehensive public health approach. The public health approach focuses on populations rather than on individuals, puts emphasis on prevention and not just on care and stresses the goal of equity and justice.

But if all this is true for Global Health why cannot be true for GMH as well? In other words, why GMH should be reduced just to making psychiatric medical treatments available to more people?

Are we not accepting reductionism rather than joining the ambitions of global health?

In occasion of its 10-year anniversary the Centre for Global Mental Health at the London School of Hygiene and Tropical Medicine organised a symposium on ‘Rebalancing Power in Global Mental Health’: the event explored the historical control of the global north in dominating narratives within research and evidence, capacity building, policy frameworks and global recommendations.

Inspired by that symposium it would be useful establishing an independent and very inclusive think tank which should promote a frank and constructive global debate on these issues and offer an unbiased support to WHO.
